# State Regulations Governing Firearms in Early Care and Education Settings in the US

**DOI:** 10.1001/jamanetworkopen.2020.3321

**Published:** 2020-04-22

**Authors:** Sara E. Benjamin-Neelon, Elyse R. Grossman

**Affiliations:** 1 Johns Hopkins Bloomberg School of Public Health, Department of Health, Behavior and Society, Johns Hopkins University, Baltimore, Maryland

## Abstract

This cross-sectional study documents existing US state regulations governing the presence and storage of firearms in early care and education settings and the extent to which these regulations align with national standards.

## Introduction

Preventing firearm-related injuries in young children is a public health priority. Firearm fatalities are the third leading cause of injury-related deaths in US children, and firearms within the family home put children at risk.^1^ Practices to mitigate this risk include storing firearms locked and unloaded, with ammunition also locked and stored separately. However, fewer than 50% of gun owners reported at least 1 safe storage practice, although households with children more often reported engaging in safe storage practices.^2^ Despite this, approximately 20% of gun-owning households with children stored firearms in an unsafe manner (ie, loaded and unlocked).^3^

Recently, there has been a substantial increase in public support for policies requiring safer storage of firearms. Child access prevention (CAP) laws are 1 example, because they may reduce firearm-related injuries.^4^ Many US states have CAP laws that govern firearms within the family home.^4^ However, nearly two-thirds of children younger than 5 years spend substantial time in out-of-home early care and education (ECE) settings.^5^ These settings are regulated by states and other jurisdictions, but to date these regulations have not been included in prior reviews of existing laws. Moreover, to our knowledge, no data are available on firearm-related injuries in ECE settings. We sought to document existing regulations governing the presence and storage of firearms in ECE settings and the extent to which state regulations align with national standards.

## Methods

Ethical approval was not required by the Johns Hopkins Bloomberg School of Public Health because human participants were not included in this regulations review. For this cross-sectional study, we reviewed ECE regulations for all 50 states, the District of Columbia, and the US territories (hereafter referred to as *jurisdictions*) through June 2019. We reviewed regulations for childcare centers (hereafter referred to as *centers*) and family childcare homes (hereafter referred to as *homes*), 2 primary types of ECE settings. Centers are typically located in dedicated buildings with more children and staff. Homes are most often located in the caregiver’s own residence with fewer children and staff. We compared regulations with 3 firearm standards put forth by the American Academy of Pediatrics, the American Public Health Association, and the National Resource Center for Health and Safety in Child Care and Early Education, including no firearms within the premises; if firearms are present they should be unloaded, equipped with protective devices, kept under lock and key, and stored separately from ammunition; and parents should be notified if firearms are on the premises.^6^ Two independent coders documented state regulations obtained from a US Department of Health and Human Services database.

## Results

Thirty-three jurisdictions had regulations prohibiting firearms on the premises for centers (Table 1), but only 7 jurisdictions prohibited firearms for homes (Table 2). Instead, many jurisdictions had regulations governing firearm storage in homes. Few jurisdictions had regulations requiring that parents be notified of firearms on the premises (2 jurisdictions for centers and 8 jurisdictions for homes). Thirteen jurisdictions did not have any regulations related to firearms for centers, and 9 jurisdictions did not have any for homes.

**Table 1.** State Regulations Consistent With National Firearm Standards for Childcare Centers Through June 2019

State or territory	No firearms within premises	If firearms are present, they should be:				Parents should be notified if firearms are on premises
		Unloaded	Equipped with protective devices	Kept under lock and key	Stored separately from ammunition	
Alabama	Yes					
Alaska						
Arizona	Yes					
Arkansas	Yes					
California	Yes			Yes		
Colorado	Yes					
Connecticut	Yes					
Delaware	Yes					
District of Columbia	Yes					
Florida	Yes					
Georgia	Yes					
Hawaii						
Idaho				Yes	Yes	
Illinois						
Indiana	Yes					
Iowa						
Kansas			Yes	Yes		
Kentucky				Yes		
Louisiana	Yes					
Maine	Yes					
Maryland	Yes	Yes		Yes		
Massachusetts						
Michigan						
Minnesota						
Mississippi	Yes		Yes	Yes		
Missouri				Yes		
Montana				Yes	Yes	
Nebraska	Yes			Yes	Yes	
Nevada	Yes					
New Hampshire				Yes		
New Jersey						
New Mexico	Yes					
New York	Yes					
North Carolina	Yes					
North Dakota			Yes	Yes	Yes	
Ohio	Yes			Yes		
Oklahoma		Yes		Yes	Yes	Yes
Oregon	Yes					
Pennsylvania	Yes					
Rhode Island	Yes					
South Carolina	Yes					
South Dakota						
Tennessee	Yes			Yes		
Texas	Yes			Yes	Yes	
Utah		Yes		Yes	Yes	
Vermont	Yes					
Virginia						
Washington	Yes					
West Virginia	Yes					
Wisconsin	Yes					
Wyoming	Yes					Yes
American Samoa						
Guam						
Northern Mariana Islands				Yes		
Puerto Rico						
US Virgin Islands	Yes					
Total	33	3	3	17	7	2

**Table 2.** State Regulations Consistent With National Firearm Standards for Family Childcare Homes Through June 2019

State or territory	No firearms within premises	If firearms are present, they should be:				Parents should be notified if firearms are on premises
		Unloaded	Equipped with protective devices	Kept under lock and key	Stored separately from ammunition	
Alabama		Yes		Yes	Yes	
Alaska						
Arizona		Yes		Yes	Yes	
Arkansas		Yes		Yes		
California			Yes	Yes	Yes	
Colorado		Yes		Yes	Yes	
Connecticut	Yes	Yes		Yes	Yes	
Delaware				Yes	Yes	
District of Columbia	Yes	Yes	Yes	Yes	Yes	Yes
Florida				Yes		
Georgia	Yes			Yes		Yes
Hawaii						
Idaho				Yes	Yes	
Illinois	Yes	Yes		Yes	Yes	Yes
Indiana				Yes		
Iowa						
Kansas				Yes	Yes	
Kentucky				Yes		
Louisiana						
Maine		Yes		Yes	Yes	
Maryland		Yes		Yes		
Massachusetts		Yes	Yes	Yes	Yes	Yes
Michigan		Yes	Yes	Yes	Yes	Yes
Minnesota		Yes		Yes	Yes	
Mississippi	Yes		Yes	Yes		
Missouri				Yes		
Montana				Yes	Yes	
Nebraska				Yes	Yes	
Nevada		Yes		Yes	Yes	
New Hampshire				Yes		
New Jersey						
New Mexico		Yes		Yes		
New York		Yes	Yes	Yes	Yes	Yes
North Carolina		Yes		Yes	Yes	
North Dakota			Yes	Yes	Yes	
Ohio				Yes		
Oklahoma		Yes		Yes	Yes	
Oregon		Yes		Yes	Yes	
Pennsylvania				Yes	Yes	Yes
Rhode Island	Yes	Yes		Yes	Yes	
South Carolina				Yes		
South Dakota						
Tennessee				Yes		
Texas				Yes	Yes	
Utah		Yes		Yes	Yes	
Vermont				Yes	Yes	
Virginia		Yes		Yes	Yes	
Washington		Yes	Yes	Yes		
West Virginia				Yes		
Wisconsin				Yes		
Wyoming		Yes		Yes	Yes	Yes
American Samoa						
Guam						
North Mariana Islands				Yes		
Puerto Rico						
US Virgin Islands	Yes					
Total	7	23	8	46	29	8

## Discussion

Although most jurisdictions did not allow firearms on the premises in centers, this was not the case for homes. Instead, these ECE settings had some regulations governing the safe storage of firearms. Despite this, most jurisdictions did not require ECE staff to notify parents if firearms were present, which could undermine parents’ ability to make informed decisions about childcare. Owners of family childcare homes, however, may own firearms for perceived safety reasons, which is a right that may be protected under the Second Amendment to the US Constitution. Additionally, nearly one-quarter of jurisdictions (13 jurisdictions) did not have any regulations governing firearms in ECE centers, and one-sixth (9 jurisdictions) did not have such regulations for homes. This raises concerns about the potential for firearm-related injuries in young children in these ECE settings.

This study has limitations. We assessed the existence but not enforcement of regulations. We also did not compare jurisdictions with CAP or other laws governing firearms with those with ECE regulations. Jurisdictions may have CAP or other laws^4^ that would apply to family childcare homes as both a home-based business and a residence. Thus, jurisdictions may not reiterate CAP or other laws in their ECE regulations. Having ECE regulations could benefit both families and communities, including medical care practitioners who deal with the aftermath of firearm-related injuries. Therefore, we recommend that US jurisdictions consider promulgating or strengthening regulations related to firearms to align with national standards.
